# Mutational effects of the asparagine198 and glutamate223 residues on the human norepinephrine transporter on basal and HIV-1 Tat protein-induced inhibition of dopamine transport

**DOI:** 10.1016/j.ejphar.2025.178131

**Published:** 2025-09-12

**Authors:** Katherine Darby Porter, Charles Adeniran, Ana Catya Jimenez-Torres, Karl Lee Pless, Abagail Brenda Cirincione, Harper Davenport, Carolyn Chen, Chang-Guo Zhan, Jun Zhu

**Affiliations:** a Department of Drug Discovery and Biomedical Sciences, College of Pharmacy, University of South Carolina, Columbia, SC, USA; b Molecular Modeling and Biopharmaceutical Center, University of Kentucky, Lexington, KY, USA; c Department of Pharmaceutical Sciences, College of Pharmacy, University of Kentucky, Lexington, KY, USA; d Department of Pharmacology and Toxicology, Medical College of Gerogia, Augusta University, Augusta, GA, USA

**Keywords:** HIV, Dopamine, Norepinephrine transporter, Uptake, Conformational transitions

## Abstract

HIV-1 transactivator of transcription (Tat) protein induces dopaminergic dysregulation, which plays a central role in HIV-1-associated neurocognitive disorders. Computational modeling predicts that asparagine 198 and glutamate 223 of the human norepinephrine transporter (hNET) are key residues involved in Tat binding. This study investigated the effects of N198A and E223A mutations on basal and Tat-induced inhibition of dopamine (DA) uptake via hNET in CHO cells expressing WT hNET or its mutants. Compared to WT hNET, E223A mutation increased the affinity for nisoxetine and cocaine in inhibiting [^3^H]DA uptake. However, N198A and E223A decreased the affinity for cocaine inhibiting [^3^H]WIN35,428 binding, without altering the [^3^H]WIN35,428 binding under control. Kinetic analysis of [^3^H]DA uptake revealed that N198A and E223A did not alter the affinity for DA uptake but reduced the maximal velocity compared to WT hNET. An optimization study using recombinant Tat_1-86_ at 0.25–140 nM revealed a K_i_ of 3.4 nM for inhibiting hNET-mediated DA uptake, with inhibition plateauing at above 8.75 nM. Treatment with 140 nM recombinant Tat_1-86_ resulted in a 34 % reduction in [^3^H]DA uptake in WT hNET, which was attenuated in the N198A mutant but remained unchanged in E223A. However, the inhibition of [^3^H]DA uptake by 8.75 nM rTat_1-86_ in WT hNET was attenuated in N198A and E223A. Moreover, N198A and E223A altered transporter conformational dynamics, as evidenced by changing the efflux of [^3^H]DA and [^3^H]MPP+. Collectively, these findings support the role of asparagine198 and glutamate223 as essential recognition residues in Tat-induced inhibition of DA uptake through hNET.

## 1. Introduction

Approximately 50 % of the 39 million HIV-1 positive individuals have neurophysiological dysfunction and HIV-associated neurocognitive disorders (HAND), which are linked to viral infection in the central nervous system (CNS), irrespective of adherence to combination antiretroviral therapy (cART) (Heaton et al., 2010). HAND encompasses a spectrum of neurocognitive deficits that range from asymptomatic or mild to HIV-associated dementia (Cross et al., 2013; Eggers et al., 2017; Horn et al., 2013; Mohamed et al., 2020; Overton et al., 2013; Saylor et al., 2016), characterized by deficits in memory, motor function, cognitive processing, and behavioral regulation (Gartner, 2000; McArthur et al., 2010; Rackstraw, 2011). Specifically, cognitive impairments are estimated to affect approximately 39.6 % of individuals living with HIV-1 (Keng et al., 2023). The CNS acts as a viral reservoir, as most cART drugs cannot effectively cross the blood-brain barrier (BBB) (Osborne et al., 2020), allowing HIV-infected macrophages to introduce viral proteins into this inadequately treated region (Buckner et al., 2006; Burdo et al., 2013; Marban et al., 2016). Replication and expression of neurotoxic viral proteins within the CNS contribute to neuropathology and the onset of HAND (Gaskill et al., 2009). The HIV-1 transactivator of transcription (Tat) protein is a key pathogenic factor in HAND, contributing to neurotoxicity and the cognitive impairments observed in affected individuals (King et al., 2006; Rappaport et al., 1999). Long-term exposure to the HIV-1 Tat protein has been shown to dysregulate the mesocorticolimbic dopamine (DA) system (Berger and Arendt, 2000; Koutsilieri et al., 2002; Nath, 2002). Neuropsychological assessments, including neuroimaging (Chang et al., 2008; Kumar et al., 2011), along with postmortem brain analysis (Gelman et al., 2012), support a link between this dopaminergic dysregulation and neurocognitive deficits observed in HAND. Uncovering the underlying molecular mechanisms of Tat-induced dopamine dysregulation is essential for advancing the development of novel therapeutic strategies for NeuroHIV.

HIV-1-induced neurocognitive impairments are characterized by an initial elevation in extracellular DA levels (Scheller et al., 2010), followed by prolonged phases of DA depletion in the brain (Kumar et al., 2009, 2011; Sardar et al., 1996). Clinical observations have shown elevated CSF DA levels in both therapy-naïve individuals with asymptomatic HIV infections (Scheller et al., 2010) and in patients receiving cART with undetectable viral loads (Horn et al., 2013). In contrast, decreased DA levels have been observed in HIV positive individuals from both the pre-cART (Berger et al., 1994; Larsson et al., 1991) and post-cART treatment eras (di Rocco et al., 2000; Obermann et al., 2009), as well as in separate postmortem brain analyses of HIV positive individuals (Kumar et al., 2009; Sardar et al., 1996). In the prefrontal cortex (PFC), a brain region essential for higher cognitive functions, the monoamine presynaptic transporters DA transporter (DAT) and norepinephrine transporter (NET) both mediate the reuptake of extracellular DA into the cytosol of the synaptic terminal (Dalley et al., 2004; Horn, 1973; Miller and Cohen, 2001; Raiteri et al., 1977; Ridderinkhof et al., 2004; Torres et al., 2003). Our previous studies have shown that the Tat protein decreases DA transport in cells expressing human DAT (Midde et al., 2013, 2015; Quizon et al., 2016) and human NET (Strauss et al., 2022). More recently, we demonstrated that DA uptake via DAT and NET is reduced in the PFC of inducible Tat_1-86_ transgenic mice (Strauss et al., 2020). Furthermore, in DA-rich brain regions such as the PFC, HIV-induced elevations in the CNS DA levels can enhance viral replication in human macrophages leading to the release of viral protein (Gaskill et al., 2017) that have been implicated in the neuropathophysiology of HAND. Therefore, elucidating the mechanistic basis of Tat interactions with DAT and NET is essential for developing novel therapeutic strategies to treat HAND and its associated comorbidities.

Using a combined computational modeling and experimental validation approach, we identified the binding mode of the Tat protein to the human NET (Adeniran et al., 2021) and demonstrated that tyrosine467 is a key residue involved in the Tat-NET interaction (Strauss et al., 2022). This study presents a refined computational model of the hNET based on a hDAT template, characterizes the effects of varying concentrations of Tat on hNET-mediated DA uptake, and investigates the roles of the identified residues involved in Tat-hNET interactions. The experimental data obtained in this study demonstrate the concentration-dependent effects of Tat and confirm its role as an allosteric modulator of the hNET, with asparagine198 and glutamate223 identified as key residues involved in Tat-hNET binding.

## 2. Materials and methods

### 2.1. Materials

Chinese hamster ovarian cells (CHO-K1, ATCC^®^ CCL-61) were purchased from American Type Culture Collection (Manassas, VA). Ham’s F-12 Medium (1X), Opti-Mem Reduced Serum Medium, and penicillin/streptomycin were purchased from Gibco Life Technologies Corporation (Grand Island, NY). Heat inactivated fetal bovine serum and trypsin/EDTA were purchased from Corning Life Sciences (Woodland, CA). Dopamine, cocaine, nisoxetine, nomifensine, desipramine, pyrocatechol, L-ascorbic acid, sodium chloride, potassium chloride, magnesium sulfate, calcium chloride, potassium dihydrogen phosphate, D-(+)-glucose, HEPES, EDTA, dithiothreitol, pargyline hydrochloride, Protease Inhibitor P8340, and Protease Inhibitor P2850 were purchased from Sigma-Aldrich (St. Louis, MO). Polyethylenimine (PEI) and Tris were purchased from Bio-Rad (Hercules, CA). [^3^H]Dopamine (3,4-ethyl-2[N-P^3^PH]dihydroxyphenylethylamine ([^3^H]DA); specific activity, 33.2 Ci/mmol), [^3^H]WIN35428 (specific activity 82.5 Ci/mmol) and [^3^H]MPP^+^ (specific activity 82.9 Ci/mmol) were purchased from PerkinElmer Life and Analytical Sciences (Boston, MA). The recombinant HIV-1 transactivator of transcription (_r_Tat_1-86_) was purchased from ImmunoDX (Woburn, MA).

### 2.2. Molecular modeling

There is currently no crystal structure of NET for any organism. To study the interaction between HIV-1 Tat and NET, the homology modeling of the HIV-1 Tat–hNET complex began by performing sequence alignment of hNET and dDAT with Mega X to generate a fasta file (Kumar et al., 2018). The fasta file was imported into Modeller to model hNET using dDAT as a template (Eswar et al., 2006). The dDAT crystal structure was obtained from RCSB PDB ID: 4XP1 (Penmatsa et al., 2013). The HIV-1 Tat crystal structure was obtained from RCSB PDB ID: 1JFW (Peloponese et al., 2000). The HIV-1 Tat NMR structure and the hNET modeled structure were prepared with the Rosetta software (Marze et al., 2018) to create 550 unique structures respectively, with the relax protocol. The Rosetta Commons suite was then used to dock HIV-1 Tat to hNET using the flexible protein protocol. The best protein-protein binding conformation was selected according to the best interface score provided by Rosetta’s software suite and then according to the distance between HIV-1 Tat M1 and hNET Y467 residues (Lemmon and Meiler, 2012).

### 2.3. Construction of plasmids

**Fig. 1.**
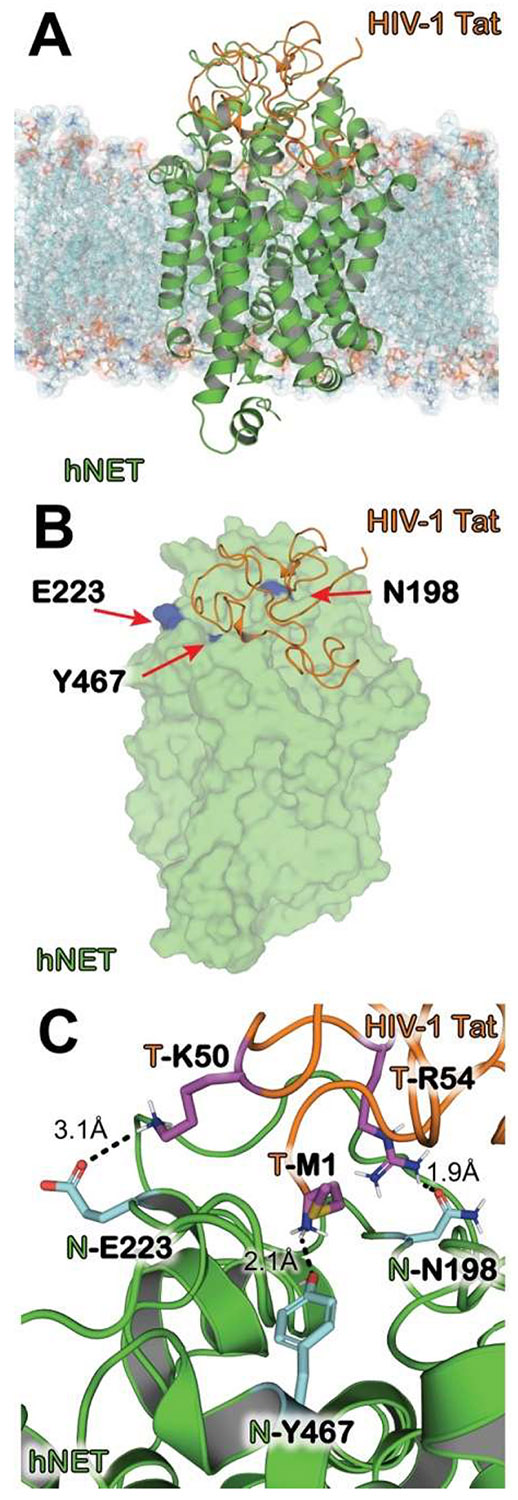
The modeled structure of HIV-1 Tat bound to hNET in the outward-open state. (A) The entire HIV-1 Tat–hNET binding complex structure developed from homology modeling. HIV-1 Tat (orange) shown with the cartoon representation, hNET (green) is represented using the cartoon representation. The lipid membrane is shown using semitransparent surface and stick representation colored according to heavy atoms. (B) Highlighting HIV-1 Tat bound to hNET with critical residues important for maintaining the formed complex. hNET (green) is shown with the surface representation showing the shape complementarity. HIV-1 Tat (orange) shown here with the cartoon representation. Amino acid residues Y467, N198, and E223 of hNET (purple) are all shown in surface representation. (C) Internuclear distances are shown between the binding interface of HIV-1 Tat–hNET binding structure. HIV-1 Tat (orange) and hNET (green) are represented in the cartoon representation. Residues Y467, N198, and E223 of hNET (cyan) as well as M1, K50, and R54 of HIV-1 Tat (magenta) shown as sticks which are important for maintaining the binding between the two proteins. Intermolecular hydrogen bonds are indicated as dashed lines, and distances are labeled next to the respective lines.

The Asparagine198 (Asn198) and Glutamic acid223 (Glu223) residues on hNET were chosen based on predictions of computational modeling and simulations (Fig. 1). Mutations of Asn198 to an alanine (N198A) and Glu223 to an alanine (E223A) are each expected to individually abolish a critical hydrogen bond between the hNET and HIV-1 Tat. Each mutant was generated based on the wild type hNET (WT hNET) sequence (NCBI, cDNA clone MGC: 190603 IMAGE: 100062757) by site-directed mutagenesis as reported previously (Strauss et al., 2022). Synthetic cDNA encoding hNET subcloned into pcDNA3.1+ was used as a template to generate mutants using the QuikChange^™^ site-directed mutagenesis Kit (Agilent Tech, Santa Clara CA). The sequences of the mutant constructs were confirmed at the University of South Carolina EnGenCore facility. DNA plasmids were propagated and purified using a Qiagen Plasmid Maxi Kit (Valencia, CA).

### 2.4. Cell culture and DNA transfection

Chinese Hamster Ovarian (CHO) Cells were grown at 37 °C in a 5 % CO_2_ incubator in 1X Ham’s F-12 Medium (Gibco Life Technologies Corporation, Grand Island, NY) supplemented with 10 % Heat-Inactivated Fetal Bovine Serum (R&D Systems) and 1 % antibiotics (100 U/ml penicillin and 100 μg/mL streptomycin). For intact cell experiments, once the cells were grown to 90 % confluency in 10 cm plates (Corning, NY), they were seeded into 24-well plates (CytoOne, USA Scientific) or precoated poly-D-lysine 24-well plates (Corning, NY) with a density of 1 × 10^5^ cells/cm^2^. 24 h after seeding, cells were transfected with WT hNET or its mutants using 0.8 μg plasmid DNA/well via Lipofectamine 2000 (Life Technologies). For cell suspension experiments, CHO cells were grown to 90 % confluency in 10 cm plates and transfected WT hNET or its mutants using 24 μg plasmid DNA/plate via Lipofectamine 2000.

### 2.5. [^3^H]DA uptake assay

To determine whether the hNET mutations alter basal substrate affinity, the competitive inhibition of [^3^H]DA was determined in WT hNET or its mutants transfected in 24-well plates based on our previous studies (Midde et al., 2013; Strauss et al., 2022). Cells were preincubated for 10 min at room temperature with a series of 10 final concentrations of DA (1 nM–1 mM), nisoxetine (0.1 nM–100 μM), or cocaine (1 nM–100 μM) in 1 x Krebs-Ringer-HEPES (KRH) buffer (final concentration in mM: 125 NaCl, 5 KCl, 1.5 MgSO_4_, 1.25 CaCl_2_, 1.5 KH_2_PO_4_, 10 D-glucose, 25 HEPES, 0.1 EDTA, 0.1 pargyline, and 0.1 L-ascorbic acid; pH 7.4). Nonspecific uptake was determined in two wells using the hNET inhibitors desipramine and nomifensine (100 μM and 10 μM final concentration, respectively). After the preincubation period, a fixed concentration of mixed [^3^H]DA (50 nM, final concentration) was added to each well and incubated for an additional 8 min at room temperature. To terminate the reaction, the reagents were removed, and cells washed twice with ice-cold 1 x KRH. Cells were lysed with 500 μL of 1 % SDS for 1 h and radioactivity measured by a liquid scintillation counter (Tri--Carb 2900 TR; PerkinElmer Life and Analytical Sciences, Waltham, MA) using disintegrations per minute (DPM) values, a standard measure of radioactivity where numerical values are proportional to the radioactivity of the sample.

The kinetic parameters of [^3^H]DA uptake, maximal velocity (V_max_) and Michalis-Menton constant (K_m_), were determined in CHO cells transiently transfected with WT hNET and its mutants seeded in 24 well plates as described above. 24 h after transfection, cells were washed with 1 x KRH buffer and preincubated for 10 min at room temperature in the presence or absence of desipramine and nomifensine (100 μM and 10 μM final concentration, respectively). After the 10-min incubation period, cells were incubated for 8 min at room temperature with a series of one of six concentrations of mixed [^3^H]DA (0.001–1.0 μM, final concentration). The reaction was terminated by the removal of solution from the wells followed by three rapid washes of ice-cold 1 x KRH. Cells were lysed with 500 μL of 1 % SDS for 1 h and radioactivity measured as described above. Specific hNET-mediated DA uptake was calculated by subtracting the nonspecific uptake (in the presence of desipramine and nomifensine) from the total uptake.

To determine whether the hNET mutants attenuate the HIV-1 Tat inhibitory effects seen in WT hNET, we performed [^3^H]DA uptake in CHO cells in the presence or absence of the Tat protein. Cells were grown to confluency in 10 cm plates then transfected with WT hNET or its mutants as described above. To prepare cell suspension, cells were detached from the 10 cm plates using trypsin/EDTA (0.25 %/0.1 %), resuspended in cell culture medium, and incubated at room temperature for 10 min. Cells were then centrifuged for 5 min at 500×*g* at 4 °C. The resulting cell pellets were washed and resuspended in phosphate-buffered saline then centrifuged at 500×*g* at 4 °C for an additional 5 min. The resulting cell pellets were then resuspended in 1 x KRH buffer. The cell suspensions from WT hNET or its mutants were then preincubated for 20 min at room temperature in the presence or absence of rTat_1-86_ (140 nM or 8.75 nM, final concentration) and the presence or absence of the nonspecific hNET inhibitors desipramine (100 μM, final concentration) and nomifensine (10 μM, final concentration). After the preincubation period, a mixed [^3^H]DA (50 nM, final concentration) was added to each sample and incubated for 8 min at room temperature. The reaction was terminated via filtration through GF/B glass filters (Brandel, Inc.) presoaked in 1 % polyethylenimine solution using three washes of ice-cold 1 x KRH buffer containing 1 mM pyrocatechol in a Brandel cell harvester (Model M-48; Brandel Inc., Gaithersburg, MD). Radioactivity was determined as described above.

To determine the inhibitor constant (K_i_) of rTat_1-86_ inhibiting DA uptake in WT hNET, cells in 10 cm plates were cultured and transfected with WT hNET as described above. All reagents were made in 1 x KRH containing 0.1 % Protease Inhibitor Cocktail 1 (P2850, Sigma-Aldrich) and 0.1 % Protease Inhibitor Cocktail (P8340, Sigma-Aldrich). Cells were detached and harvested as described above. The cell suspensions were then incubated with a range of rTat_1-86_ (0.25–140 nM, final concentrations) for 20 min at room temperature. Mixed [^3^H]DA (50 nM, final concentration) was added to each sample and incubated for 8 min at room temperature. The reaction was then terminated as described above.

### 2.6. [^3^H]WIN35428 binding assay

To determine whether the hNET mutations alter basal binding potencies of substrate and inhibitors, the competitive inhibition of [^3^H] WIN35428 binding was determined in WT hNET or its mutants transfected in precoated 24-well plates. Cells were incubated at room temperature with a series of 10 final concentrations of cocaine (1 nM–100 μM) or nisoxetine (0.01 nM–10 μM) in sucrose-phosphate buffer (7.3 mM Na_2_HPO_4_ 7H_2_O anhydrous, 2.1 mM NaH_2_PO_4_ H_2_O, and 320 mM sucrose) and a fixed concentration of [^3^H]WIN35428 (5 nM, final concentration) for 15 min. Nonspecific uptake was determined using desipramine (100 μM, final concentration). After the 15-min incubation period, the reagents were removed, and wells washed twice with ice-cold sucrose-phosphate buffer. Cells were lysed with 1 % SDS, and radioactivity determined as described above.

### 2.7. Basal [^3^H]DA and [^3^H]MPP^*+*^ efflux assays

To determine whether N198A or E223A hNET alters transporter conformational transients, [^3^H]DA or [^3^H]MPP^+^ efflux was performed based on our previous studies (Midde et al., 2015; Quizon et al., 2021). Intact CHO cells in precoated 24-well plates transfected with WT hNET or its mutants were preloaded with [^3^H]DA (50 nM final concentration) for 20 min or [^3^H]MPP^+^ (5 nM final concentration) for 30 min at room temperature. After the preloading period, cells were washed twice with 1 x KRH buffer before collecting fractional release samples. Total uptake of [^3^H]DA or [^3^H]MPP^+^ at the zero time point was estimated from four wells per genotype that were lysed immediately with 1 % SDS after preloading period. Nonspecific uptake of [^3^H]DA or [^3^H]MPP^+^ at zero time point was estimated from four wells per genotype that were incubated with the hNET inhibitors desipramine and nomifensine (100 μM and 10 μM final concentration, respectively) and lysed immediately with 1 % SDS after the preloading period. To collect fractional release, 500 μL of KRH buffer was added into a set of four wells and transferred to scintillation vials after 0 min as the initial fractional efflux. Fresh 500 μL of 1 x KRH buffer was added into the wells and incubated for 10 min then transferred to scintillation vials. Fractional efflux was collected every 10 min for 90 min ([^3^H]DA) or 110 min ([^3^H]MPP^+^). After the last fractional efflux was collected, the cells were lysed with 1 % SDS and samples collected as leftover [^3^H]DA or [^3^H]MPP^+^ remaining in the cells.

### 2.8. Statistical analysis

Results are presented as mean ± SEM. The number of n represents the number of independent experiments from each experiment group. Kinetic parameters (V_max_ and K_m_) were determined from saturation curves by nonlinear regression analysis using a one-site model with variable slope. IC_50_ values for the substrate and inhibitors for [^3^H]DA uptake were determined from nonlinear regression analysis inhibition curves using a one-site model with variable slope. GraphPad Prism (version 10.1.2.) was used to calculate the kinetic parameters and IC_50_ values for substrate and inhibitors inhibiting [^3^H]DA uptake or [^3^H] WIN35428. For any experiment involving comparisons between unpaired samples, unpaired Student’s *t* tests were used to determine any difference between parameters between WT hNET and its mutants. Log-transformed IC_50_ values were used for statistical comparisons. One-way ANOVA comparisons with repeat measurements were used for analyzing the [^3^H]DA and [^3^H]MPP^+^ efflux data. Significant differences between samples were analyzed with independent ANOVAs followed by post-hoc tests, which is indicated in the Results Section of the efflux experiments. All statistical analyses were performed using the IBM SPSS Statistics version 29 and returned values of at least *p* < 0.05 were considered significant.

## 3. Results

### 3.1. Computational modeling

Fig. 1 shows the modeled HIV-1 Tat–hNET complex and the binding interface highlighting the residues involved in maintaining the interaction between the two proteins. Fig. 1A shows that HIV-1 (orange) binds to hNET (green) outward-open confirmational state, with the entire complex embedded in a transmembrane lipid POPC bilayer. Fig. 1B shows the excellent complementarity of HIV-1 Tat (orange) in the extracellular domain of hNET (green) due to electrostatic attraction. hNET residues Y467, N198, and E223 are highlighted in purple. These amino acid residues were predicted to be important in the interaction between HIV-1 Tat and hNET. The most critical interactions are shown with distances indicated with dashed lines in Fig. 1C and include HIV-1 Tat M1, R54, and K50 and hNET residues Y467, N198, and E223. The oxygen atom of the hydroxyl group of hNET Y467 side chain Å forms a hydrogen bonding interaction with the positively charged backbone N atom of HIV-1 Tat M1 at 2.1 Å. The carbonyl oxygen atom from hNET N198 side chain forms a hydrogen bond (HB) interaction with the N atom of the guanidinium group from HIV-1 Tat R54 at 1.9 Å. Lastly, there is a weak HB interaction between the carbonyl oxygen atom of hNET E223 side chain to the positively charged nitrogen atom of the butylammonium group from HIV-1 Tat K50 side chain at 3.1 Å. These hydrogen bonding interactions along with the electrostatic complementarity help to maintain this bound complex. Based on the modeled HIV-1 Tat–hNET binding mode depicted in Fig. 1, in addition to Y467, hNET residues N198 and E223 are also involved in the HIV-1 Tat–hNET binding, predicting that the amino-acid change on either N198 or E223 may significantly attenuate the HIV-1 Tat–hNET binding. Hence, we designed the hNET mutants N198A and E223A that were all predicted to significantly attenuate the HIV-1 Tat–hNET binding.

### 3.2. Mutational effects of Asn198 and Glu223 residues on basal NET uptake kinetic parameters and the inhibitory potency of NET-mediated DA uptake and NET binding by substrates and inhibitors

**Fig. 2.**
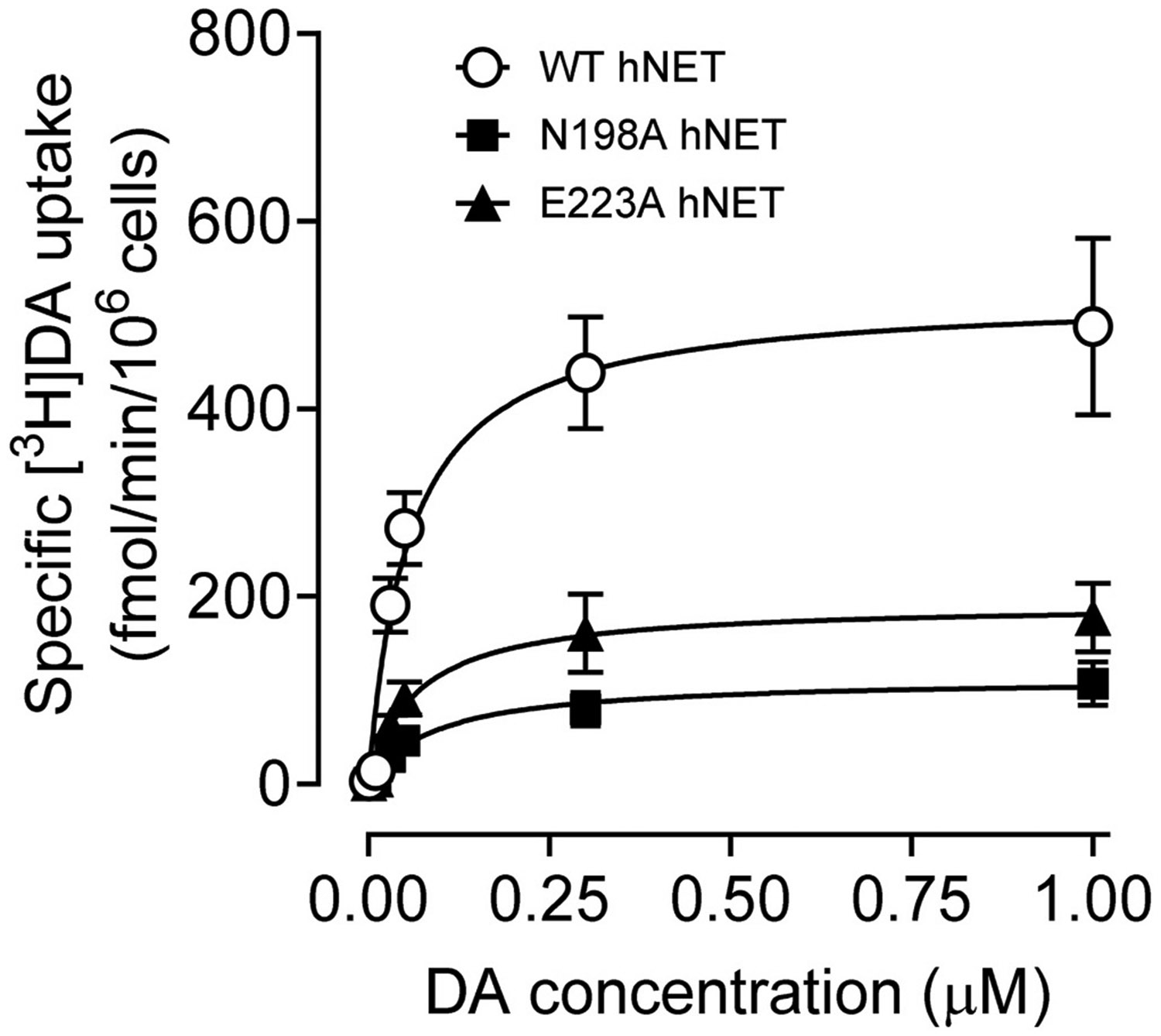
Kinetic parameters of [^3^H]DA uptake in WT hNET and mutants. The specific [^3^H]DA uptake was determined in intact CHO cells expressing WT hDAT, N198A, and E223A using six concentrations of DA (0.001–1.0 μM, final concentration) mixed with a fixed concentration of [^3^H]DA (500,000 dpm/well, specific activity: 21.2 Ci/mmol). In parallel, nonspecific uptake of each concentration of [^3^H]DA in the presence of 100 μM desipramine and 10 μM nomifensine were subtracted from total uptake for calculating the specific NET-mediated uptake. The V_*max*_ and K_*m*_ values were calculated by fitting the data to the Michaelis-Menten equation and represent the means from five to ten independent experiments ± S.E.M.

To determine the functional influence of the Asn198 and Glu223 mutants on basal hNET function, kinetic analysis of [^3^H]DA uptake was performed in CHO cells expressing WT hNET or its mutants. As shown in Table 1 and Fig. 2, compared to WT hNET (525.4 ± 89.8 fmol/min/10^6^ cells), the V_max_ values were reduced in both the N198A [91.4 ± 16.4 fmol/min/10^6^ cells, (t_(8)_ = 4.8; *p* < 0.01, unpaired Student’s *t*-test)] and E223A [151.1 ± 14.5 fmol/min/10^6^ cells (t_(8)_ = 4.1; *p* < 0.01, unpaired Student’s *t*-test)] hNET mutants by 82.6 % and 71.2 %, respectively. However, compared to WT hNET (K_m_: 0.057 ± 0.008 μM) the K_m_ values remained unchanged in both the N198A (0.084 ± 0.020 μM) and E223A (0.066 ± 0.011 μM) hNET mutants.

**Fig. 3.**
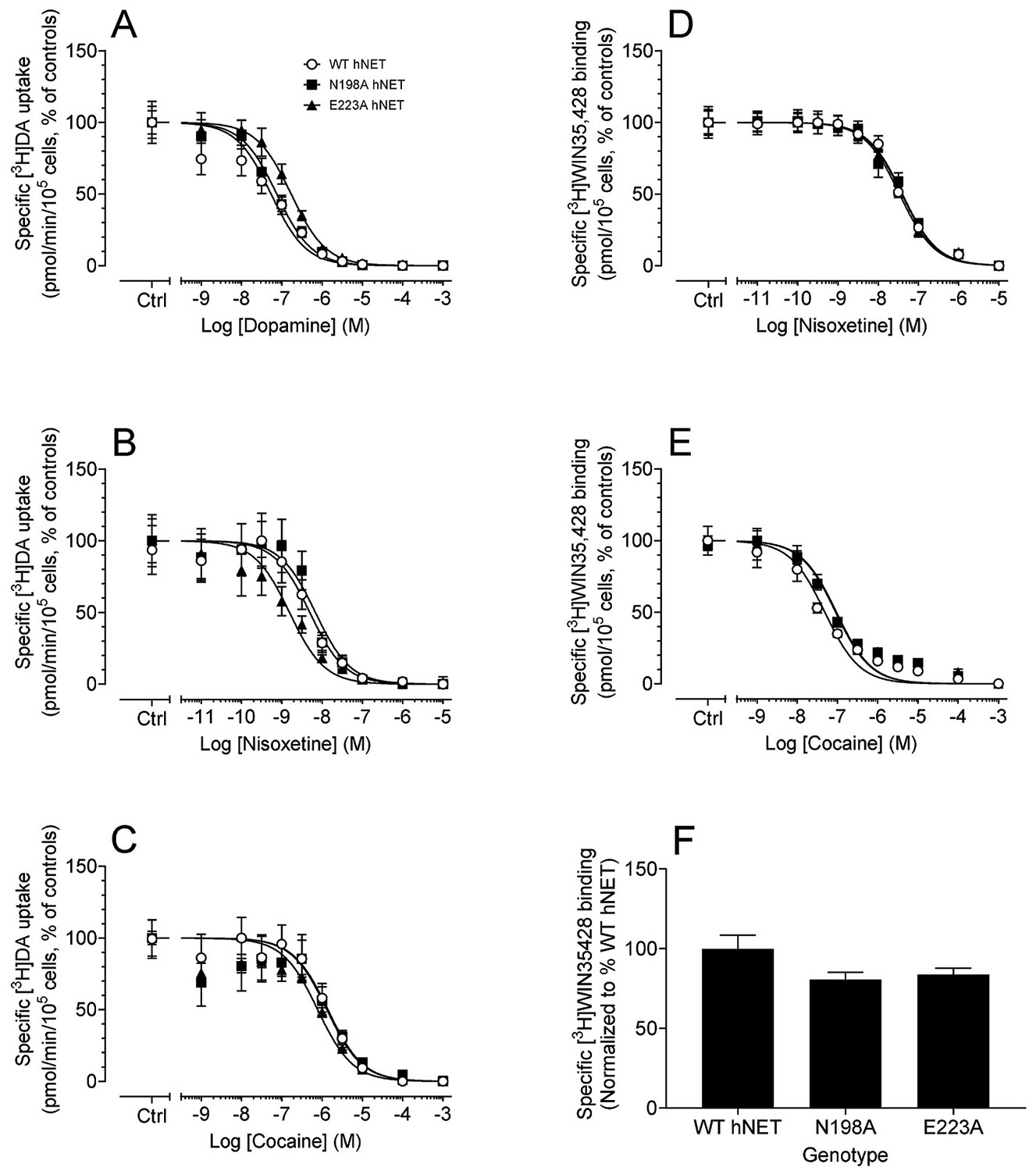
Half maximal inhibitory curves of substrate and inhibitors inhibiting [^3^H]DA uptake or [^3^H]WIN35428 binding in WT hNET and its mutants. For [^3^H]DA uptake, CHO cells expressing WT hNET or N198A and E223A seeded in 24-well plates were preincubated with a range of 10 concentrations of DA (1 nM–1 mM), nisoxetine (0.1 nM–100 μM), or cocaine (1 nM–100 μM) at a room temperature for 10 min followed by addition of a fixed [^3^H]DA (50 nM, final concentration) for additional 8 min. Half maximal inhibitory concentrations for DA and inhibitors inhibiting [^3^H]DA uptake were analyzed by nonlinear regression of the direct counts (DPM values) of [^3^H]DA from a range of concentrations relative to the respective controls for (A) DA, (B) nisoxetine, and (C) cocaine. For [^3^H]WIN35428 binding, CHO cells expressing WT hNET or N198A and E223A seeded in precoated 24-well plates were incubated with a range of 10 concentrations of nisoxetine (0.1 nM–100 μM) or cocaine (1 nM–100 μM) and a fixed [^3^H]WIN35428 (5 nM, final concentration) at a room temperature for 15 min. Half maximal inhibitory concentrations for DA and inhibitors inhibiting [^3^H]WIN35428 binding were analyzed by nonlinear regression of the direct counts (DPM values) of [^3^H]WIN35428 from a range of concentrations relative to the respective controls for (D) nisoxetine and (E) cocaine. Concentration response curves of DA and inhibitors inhibiting [^3^H]DA uptake or [^3^H]WIN35428 binding plotted on a logarithmic scale. The control values of the specific [^3^H]WIN35428 binding collected from panels D and E were presented as mean ± SEM from 10 independent experiments (F).

To determine whether mutations of hNET alter the apparent affinity of DA or inhibitors, the IC_50_ values for DA, nisoxetine or cocaine inhibiting [^3^H]DA uptake were examined in WT hNET and its mutants. As shown in Table 2 and Fig. 3, compared to WT hNET (143.4 ± 15 nM), the IC_50_ value for DA inhibiting [^3^H]DA uptake showed no differences in N198A (95.3 ± 22 nM) or E223A (165.5 ± 16 nM). In addition, compared to WT hNET (6.1 ± 1.1 nM), the IC_50_ value for nisoxetine inhibiting [^3^H]DA was decreased in E223A [2.6 ± 0.9 nM, t_(9)_ = 2.4; *p* < 0.05, unpaired Student’s *t*-test], while no change were observed in N198A (7.6 ± 1.0 nM). Compared to WT hNET (1993 ± 198 nM), the IC_50_ values for cocaine inhibiting [^3^H]DA uptake were decreased in E223A (1282 ±95 nM, *t*_(10)_ = 2.82; *p* < 0.05) but not altered in N198A (1906 ± 158 nM). To further determine whether the influence of hNET mutants on the affinity of NET-mediated DA uptake by inhibitors is associated with their affinity of NET binding sites, the IC_50_ values for nisoxetine or cocaine inhibiting [^3^H]WIN35428 binding were tested. As shown in Table 2 and Fig. 3, compared to WT hNET (25.4 ± 3.7 nM), the IC_50_ value for nisoxetine inhibiting [^3^H]WIN35428 binding showed no difference in both N198A (29.8 ± 5.0 nM) and E223A (24.9 ± 2.85 nM) mutants. Compared to WT hNET (35.5 ± 4.3 nM), the IC_50_ value for cocaine inhibiting [^3^H]WIN35428 binding was increased in both N198A hNET [72.2 ± 10.7, (t_(7)_ = 2.9; *p* < 0.05, unpaired Student’s *t*-test)] and E223A hNET [67.4 ± 6.18 (t_(7)_ = 4.0; *p* < 0.01, unpaired Student’s *t*-test)]. To evaluate whether the [^3^H]WIN35428 binding sites were altered among WT hNET and the two mutants, the control values of [^3^H] WIN35428 binding from the competitive inhibitory experiments for nisoxetine (Fig. 3D) and cocaine (Fig. 3E) were combined. As shown in Fig. 3F, specific binding of 5 nM [^3^H]WIN35428 in WT hNET or its mutants was assessed over a 15 min incubation period. The specific [^3^H] WIN35428 binding was calculated by subtracting the nonspecific binding DPMs (in the presence of 100 μM desipramine) from the total binding DPMs then normalized as a percentage of WT hNET. Compared to WT hNET, (100 ± 8.5 %, DPM: 14083 ± 1198) no differences in specific [^3^H]WIN35428 binding was observed in N198A hNET (80.67 ± 4.6 %. DPM: 11361 ± 652) and E223A hNET (83.83 ± 4.0, DPM: 11806 ± 567).

### 3.3. Concentration dependent effects of Tat protein on hNET-mediated DA uptake in WT hNET and its mutants

**Fig. 4.**
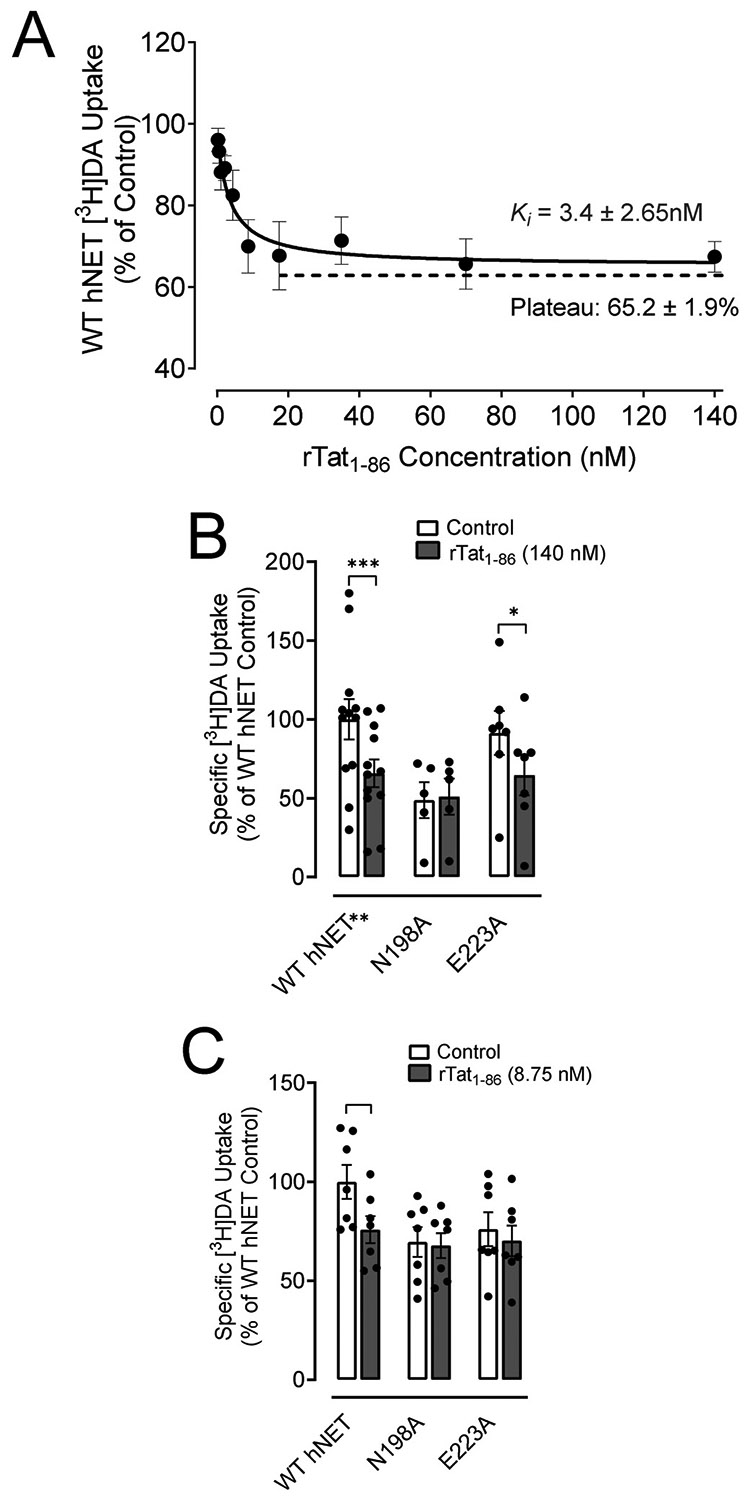
Differential inhibitory effects of rTat_1–86_ are observed on WT hNET and its mutants. (A) The inhibition constant (K_i_) value for a range of rTat_1-86_ inhibiting DA uptake through WT hNET. Data is expressed as mean ± SEM from 4 independent experiments. (B) The inhibitory effects of 140 nM rTat_1-86_ on DA uptake via WT hNET and its mutants was determined and is presented as the ratio of the direct counts (DPM values) of [^3^H]DA uptake for each mutant in the presence of rTat_1-86_ compared to their respective control values (DPM) of [^3^H]DA uptake in the absence of Tat. [The direct counts in DPM: WT hNET without Tat 1870 ± 238, with Tat: 1230 ± 163, N198A hNET without Tat: 912 ± 212, with Tat: 954 ± 213, E223A hNET without Tat: 1711 ± 261, with Tat: 1206 ± 239]. Data is expressed as mean ± SEM from 11 independent experiments (WT hNET) and 5–6 independent experiments (mutants). *p < 0.05 compared to control value (in the absence of Tat, paired Student’s *t*-test), ***p < 0.001 compared to control value (in the absence of Tat, paired Student’s *t*-test). (C) The inhibitory effects of 8.75 nM rTat_1-86_ on DA uptake via WT hNET and its mutants was determined and is presented as the ratio of the direct counts (DPM values) of [^3^H]DA uptake for each mutant in the presence of rTat_1-86_ compared to their respective control values (DPM) of [^3^H]DA uptake in the absence of Tat. Data is expressed as mean ± SEM from 7 independent experiments. [The direct counts in DPM: WT hNET without Tat: 2778 ± 240, with Tat: 2106 ± 191, N198A hNET without Tat: 1934 ± 210, with Tat: 1883 ± 175, E223A hNET without Tat: 2113 ± 237, with Tat: 1950 ± 215] **p < 0.01 compared to control value (in the absence of Tat, paired Student’s *t*-test).

To determine whether rTat_1-86_ inhibits basal NET-mediated [^3^H]DA uptake in a concentration-dependent manner, the optimization study revealed a 3.4 nM (SEM: 2.65 nM) the inhibitory constant (*K*_i_) value for Tat inhibiting the hNET-mediated DA uptake (Fig. 4A). Furthermore, the Tat’s inhibitory effect reached a 65.2 % (SEM: 1.9 %) plateau from Tat concentrations from 8.75 nM to 140 nM. Our previous studies have demonstrated that Tat interacts with hDAT in an allosteric modulatory manner (Zhu et al., 2011). Based on our computational modeling predictions, each hNET mutation is expected to individually attenuate Tat-induced inhibition of DA uptake. N198A and E223A are expected to impair hydrogen bonding interactions between Tat and hNET, leading to an attenuation of Tat-induced DA uptake. In this study, to determine whether Tat interaction with hNET is concentration dependent, two concentrations (8.75 and 140 nM) of rTat_1-86_ were chosen for testing the inhibitory effects on DA uptake in WT hNET and two mutants. As shown in Fig. 4B, addition of 140 nM rTat_1-86_ induced a 34.22 % (SEM: 8.70 %) reduction of the specific [^3^H]DA uptake in WT hNET relative to the control [without Tat: 1870 ± 238 DPM, with Tat: 1230 ± 163 DPM, (*t*_(11)_ = 6.1; *p* < 0.001, paired Student’s *t*-test)]. 140 nM Tat significantly decreased [^3^H]DA uptake by 26.98 % (SEM: 12.75 %) in E223A compared to the control [without Tat: 1711 ± 261 DPM, with Tat: 1206 ± 239 DPM, (*t*_(6)_ = 4.3; *p* < 0.01, paired Student’s *t*-test)]. However, the Tat-induced reduction of [^3^H]DA in WT hNET was attenuated in N198A compared to its control [without Tat: 912 ± 212 DPM, with Tat: 954 ± 213 DPM, (*t*_(4)_ = 1.0; *p* > 0.05, paired Student’s *t*-test)], suggesting that the mutation of N198 attenuates Tat’s inhibitory effect on DA uptake at 140 nM and that the N198 residue on hNET is involved in HIV-1 Tat-mediated inhibition of DA uptake.

We further determined whether the lower concentration (8.75 nM) of rTat_1-86_ differentially affects DA uptake through the N198A and E223A mutants. As shown in Fig. 4C, the specific [^3^H]DA uptake in the presence of 8.75 nM rTat_1-86_ was decreased in WT hNET by 24.2 % (SEM: 6.86 %) relative to the control [without Tat: 2778 ± 240 DPM, with Tat: 2106 ± 191 DPM, (*t*_(6)_ = 5.2; *p* < 0.01, paired Student’s *t*-test)]. This Tat-induced reduction of [^3^H]DA in WT hNET was attenuated in N198A compared to its control (without Tat: 1934 ± 210 DPM, with Tat: 1883 ± 175 DPM) and in E223A compared to its control (without Tat: 2113 ± 237 DPM, with Tat: 1950 ± 215 DPM).

### 3.4. Mutational effects of hNET on basal [^3^H]DA and [^3^H]MPP^*+*^ efflux

**Fig. 5.**
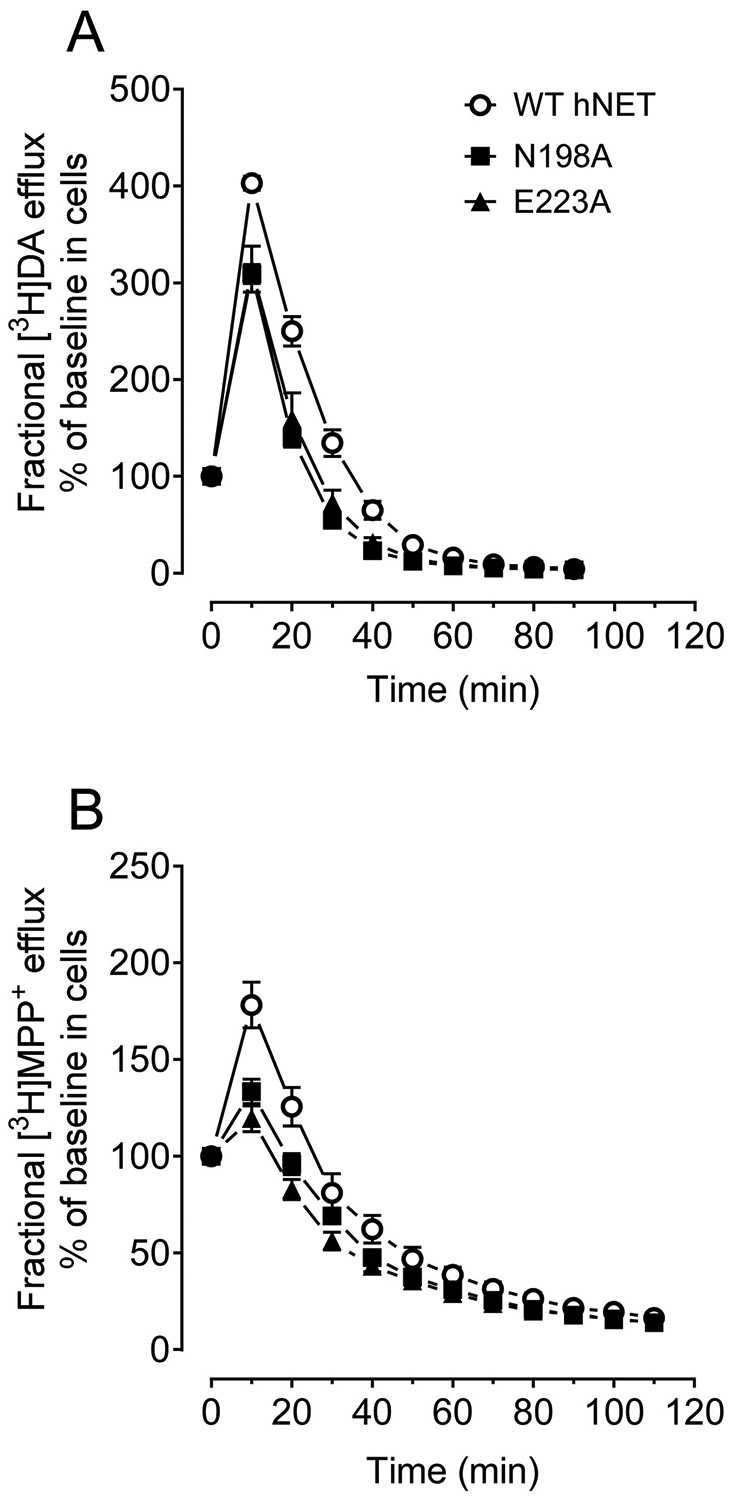
Mutational effects of hNET mutants on functional efflux of DA or MPP^þ^ . (A) [^3^H]DA efflux expressed as a percentage of baseline release after the 20-min preloading period. (B) [^3^H]MPP^+^ efflux plotted as a percentage of baseline release after the 30-min preloading period. CHO cells transfected with WT or mutants were preincubated with KRH buffer containing [^3^H]DA or [^3^H] MPP^+^ at room temperature for 20 and 30 min, respectively. After incubation, cells were washed and incubated with fresh buffer at indicated time points. Subsequently, the buffer was removed from cells, and radioactivity in the buffer and residual radioactivity in the cells was counted. Each fractional efflux of [^3^H]DA or [^3^H]MPP^+^ in WT or mutants was expressed as a percentage of total [^3^H]DA or [^3^H]MPP^+^ in the cells at the start of the experiment. Fractional efflux levels are expressed at 1–90 min for [^3^H]DA and 1–110 min for [^3^H]MPP^+^.

To determine whether the hNET Asn198 and Glu223 interacts with Tat protein through changing transporter conformational transitions, we examined the fractional efflux levels of [^3^H]DA and [^3^H]MPP^+^ in CHO cells transfected with WT hNET and the two mutants. As shown in Fig. 5, after the 20-min preloading period with 50 nM [^3^H]DA and the 30-min preloading period with 5 nM [^3^H]MPP^+^, cells were washed and initial fractional efflux samples were immediately collected as 0 time point. The efflux peaks of [^3^H]DA and [^3^H]MPP^+^ were observed at 10 min in WT hNET and its mutants. With regards to the efflux of [^3^H]DA uptake (Fig. 4A), a two-way ANOVA revealed significant main effects of mutation (F_(2, 12)_ = 13.2; *p* < 0.001), time (F_(1, 12)_ = 636; *p* < 0.001), and time × mutation (F_(18, 108)_ = 9.7, *p* < 0.001) for the two mutants when compared to WT hNET. Post-hoc analysis with a simple comparison for N198A hNET vs. WT hNET revealed significant main effects of mutation (F_(1, 8)_ = 36, *p* < 0.001), time (F_(9, 72)_ = 974, *p* < 0.001), and time × mutation (F_(9, 72)_ = 35, *p* < 0.001), suggesting that overall [^3^H]DA efflux in N198A was significantly reduced across 90 min compared to WT hNET. Post-hoc analysis with a simple comparison for E223A hNET vs. WT hNET revealed significant main effects of mutation (F(1, 8) = 12.2, *p* < 0.01), time (F_(9, 72)_ = 352, *p* < 0.001), and time × mutation (F_(9, 72)_ = 9.0, *p* < 0.001), suggesting that overall [^3^H]DA efflux in E223A was significantly reduced across 90 min compared to WT hNET. Regarding the [^3^H]MPP + efflux (Fig. 4B), a two-way ANOVA revealed significant main effects of mutation (F_(2, 15)_ = 6.5; *p* < 0.01), time (F_(11, 165)_ = 508; *p* < 0.001), and time × mutation (F_(22, 165)_ = 8.0, *p* < 0.001) for the two mutants when compared to WT hNET. Post-hoc analysis with a simple comparison for N198A hNET vs WT hNET revealed significant main effects of mutation (F_(1, 10)_ = 4.9, *p* < 0.05), time (F_(11, 110)_ = 319, *p* < 0.001), and time × mutation (F_(11, 110)_ = 6.7, *p* < 0.001), suggesting that overall [^3^H]MPP^+^ efflux in N198A was significantly reduced across 110 min. Post-hoc analysis with a simple comparison for E223A hNET vs WT hNET revealed significant main effects of mutation (F_(1, 10)_ = 9.6, *p* < 0.05), time (F_(11, 110)_ = 290, *p* < 0.001), and time × mutation (F_(11, 110)_ = 12.0, *p* < 0.001) suggesting that overall [^3^H]MPP^+^ efflux in E223A was significantly reduced across 110 min. This data supports our findings that the N198A and E223A mutations alter the Tat binding sites on hNET through a change in the transporter conformation ability, and additionally suggest that the N198A and E223A mutants reduce the capability of the transporter to undergo efficient conformational transitions.

## 4. Discussion

Our previously published work demonstrated that *in vivo* expression of HIV-1 Tat protein in the brains of inducible Tat transgenic mice reduces DA uptake through both DAT and NET in the PFC (Strauss et al., 2020). These findings suggest that in the PFC, the NET mediates the reuptake of both DA and norepinephrine, and that Tat protein-induced dysregulation of dopaminergic transmission may result from Tat-induced inhibition of both DAT and NET. Previous computational modeling of HIV-1 Tat-binding to hNET revealed that tyrosine 467 plays a critical role in mediating Tat-induced inhibition of NET function and associated conformational changes (Adeniran et al., 2021; Strauss et al., 2022). Given that Tat protein interacts with multiple NET residues, a comprehensive understanding of the Tat-NET binding interface is essential to elucidate the molecular mechanisms underlying the Tat-hNET complex. Therefore, to gain deeper insight into the Tat-hNET interaction, we further refined our computational model to identify additional contributing residues. We identified that the carbonyl oxygen atom from hNET Asn198 side chain forms a hydrogen bond interaction with the N atom of the guanidinium group from HIV-1 Tat R54 at 1.9 Å, whereas a weak hydrogen bond interaction between the carbonyl oxygen atom of hNET Glu223 side chain and HIV-1 Tat K50 side chain at 3.1 Å. Mutation of either Asn198 or Glu223 may disrupt the interactions between hNET and Tat. The modeled binding structures of DAT or NET-Tat interaction have been validated as a feasible approach for identifying targets of these transporter for Tat binding by our published studies that were focused on the transporters (DAT and NET) binding with HIV-1 Tat (Adeniran et al., 2021; Yuan et al., 2015, 2016).

The present results show that mutation of Asn198 leads to an 82.6 % reduction in the V_max_ of [^3^H]DA uptake, without affecting the K_m_ value relative to WT hNET and with no change in the IC_50_ value for DA inhibiting [^3^H]DA uptake. The Glu223 mutation resulted in a 71.2 % reduction in the V_max_ of [^3^H]DA uptake without altering the K_m_ value or the IC_50_ value for DA inhibiting [^3^H]DA uptake compared to WT hNET. These findings suggest that both N198A and E223A mutants retain basal substrate affinity but exhibit a reduced capacity for substrate uptake, likely due to impaired transporter conformational transitions necessary for efficient DA influx. Although the current results from control-specific [^3^H]WIN35428 binding sites do not indicate altered NET surface expression, they enable further refinement of computational modeling to map the Tat-hNET interaction structure, facilitating the development of allosteric probes aimed at attenuating Tat-induced dysregulation of NET-mediated dopaminergic transmission.

The present results show that the potency of nisoxetine and cocaine in inhibiting NET-mediated DA uptake was increased in E223A mutant but remained unchanged in N198A compared to WT hNET. Nisoxetine functions as a non-competitive inhibitor of NET-mediated reuptake of norepinephrine or DA (Reith et al., 2005; Zhen et al., 2012), whereas cocaine acts as a competitive inhibitor of the hNET with respect to the norepinephrine and DA uptake site (Chen and Justice, 1998). The potency of nisoxetine or cocaine may be modulated by conformational transitions of the NET. Our computational model of the Tat-hNET interaction predicts that the hNET residue Glu223 recognizes a Tat binding sites on hNET, potentially modulating hNET-mediated DA uptake through an allosteric mechanism. Thus, the E223A-induced alteration of the potency of nisoxetine and cocaine may result from conformational changes in hNET that binding sites for both inhibitors. On the other hand, with respect to [^3^H]WIN35428 binding potency, both N198A and E223A mutations do not affect the potency of nisoxetine but reduce the potency of cocaine relative to WT hNET. Nisoxetine preferentially binds to the S2 site of the hNET, whereas cocaine interacts with the S1 binding pocket (Andersen et al., 2015). WIN35428, a cocaine analog, also targets the S1 site and exhibits preferential binding to hNET in its outward-open conformation (Norregaard et al., 1998; Zhen et al., 2012). Our computational modeling predicts that both Asn198 and Glu223 serve as allosteric modulatory sites for Tat binding on hNET. Therefore, these results suggest that each mutant may independently alter the conformation of the S1 binding site, while leaving the S2 binding site, and thus [^3^H]WIN35428 binding unchanged. Collectively, studying Tat-NET binding structure provides a critical molecular framework for the development of small-molecule therapeutic targeting HAND. Notably, we have identified a novel allosteric modulator, SRI 32743, which mitigates Tat-inhibited DA uptake via both DAT and NET and alleviates Tat-potentiated cocaine reward in inducible Tat transgenic mice (Davis et al., 2023; Jimenez-Torres et al., 2024; Zhu et al., 2022;).

Our current results demonstrate a Ki value of 3.4 nM for Tat inhibition of hNET-mediated DA uptake. This concentration closely approximates the physiological levels of Tat (5–35 ng/mL) reported in the cerebrospinal fluid samples and brains of HIV-infected individuals (Johnson et al., 2013; Westendorp et al., 1995; Xiao et al., 2000). Interestingly, we observed that Tat-induced inhibition of Net-mediated DA uptake reaches a plateau at approximately 8.75 nM Tat. Given the proposed allosteric modulation of hNET, one possible mechanism is that at low concentration (8.75 nM), Tat initiates its inhibitory effect in a concentration-independent manner. This could result in a maximal inhibitory effect being reached early, with no further increase in inhibition observed up to 140 nM Tat. To support this, our previous studies demonstrated that Tat preferentially binds to the outward-open conformation of DAT and inhibits DA uptake by interacting with allosteric binding site(s) on human DAT, rather than the primary DA binding site (Yuan et al., 2015; Zhu et al., 2011). Our computational modeling predicted that mutations of N198 and E223 attenuate Tat-induced inhibition of DA uptake by disrupting Tat-hNET interactions. To validate this, we assessed NET-mediated DA uptake in the presence of rTat_1-86_ at either 8.75 nM (approximately 2.6-fold the Ki, predicted to occupy ~72% of hNET molecules) or 140 nM (approximately 41.2-fold the Ki, predicted to occupy ~98 % of hNET molecules). At a high concentration of rTat_1-86_ (140 nM), only N198A attenuated the Tat-induced decrease in DA uptake observed in WT hNET. Interestingly, at a low rTat_1-86_ concentration (8.75 nM), both N198A and E223A attenuated the Tat-induced reduction of DA transport observed in WT hNET. This is because, at the higher the Tat concentration, it becomes more difficult for a given mutation to significantly attenuate the Tat-hNET binding. Based on this theory, at 140 nM Tat, the interaction between Tat and hNET becomes more stable, making it difficult for the E223A mutation to disrupt it. In contrast, under low Tat concentration, both N198A and E223A can effectively interfere with the Tat-hNET interaction. Indeed, the N198 residue interacts with the Tat protein at a distance of 1.9 Å, while the E223 residue interacts at 3.1 Å, which may underlie the differential ability of these mutants to attenuate the Tat-induced reduction in DA uptake observed in WT hNET. These findings further support a mechanism in which hNET mutations mitigate Tat-induced inhibition of NET-mediated DA uptake by inducing conformational transitions in the transporter. Notably, in addition to our current finding that Tat inhibits hNET-mediated DA uptake *in vitro*, we have also demonstrated that *in vivo* Tat expression in inducible Tat transgenic mice for 7 or 14 days reduces DAT- and NET-mediated DA and NE uptake, respectively, which may contribute to the neurocognitive impairments observed in both animals and humans (Davis et al., 2023; Strauss et al., 2020; Zhu et al., 2022).

An important finding from the current study is that both N198A and E233A mutations significantly reduced the functional efflux of [^3^H]DA or [^3^H]MPP^+^ compared to WT hNET. Transporter-mediated efflux of [^3^H]DA or [^3^H]MPP^+^ is commonly used to assess transporter conformational changes induced by substrates or inhibitors (Johnson et al., 1998; Rothman et al., 2012). While both DA and MPP^+^ are known hNET substrates, MPP^+^ exhibits reduced membrane permeability relative to DA (Buck and Amara, 1994; Scholze et al., 2000). By examining [^3^H]DA or [^3^H]MPP^+^ efflux, this study aimed to elucidate the mechanism by which hNET mutations attenuate mediated Tat-induced inhibition of transporter function. Given that Tat modulates DAT or NET function allosterically, Asn198 and Glu223 are predicted to be key residues involved in Tat binding via conformational transitions in hNET. Supporting these findings, our previous work demonstrated that mutation of the analogous DAT residue, tyrosine 470, reduces efflux of both DA and MPP^+^ (Midde et al., 2015). Together, these results underscore the critical role of Asn198 and Glu223 in the allosteric modulation of hNET by HIV-1 Tat protein.

## 5. Conclusions

Our findings provide novel mechanistic evidence that Tat modulates hNET-mediated DA transport in a concentration-dependent manner, mediated through conformational changes in the transporter. Through computational modeling and experimental validation, we demonstrate that Asn198 and Glu223 are critical residues for Tat-induced inhibition of NET function, as mutations at these sites attenuate Tat’s effects. Importantly, investigating the functional relevance of these key NET residues in the Tat-NET interaction offers valuable insights for the development of allosteric modulators with potential therapeutic applications in HAND.

## Figures and Tables

**Table 1:** Summary of kinetic properties of [^3^H]DA uptake in WT hNET and its mutants.

	WT hNET	N198A	E223A
V_max_ (fmol/min/10^6^)	525.4 ± 89.8	91.4 ± 16.4**	151.1 ± 14.5**
K_m_ (μM)	0.057 ± 0.008	0.084 ± 0.020	0.066 ± 0.011

Data is presented as mean ± S.E.M. values from five to six independent experiments. **p < 0.01, unpaired student’s *t*-test compared to WT hNET.

**Table 2:** Summary of half-maximal inhibitory concentration values of substrates and inhibitors for inhibiting [^3^H]DA uptake and [^3^H]WIN35428 binding in WT hNET and its mutants.

	WT hNET	N198A	E223A
[^3^H]DA Uptake IC_50_ (nM)			
DA	143.4 ± 15	95.3 ± 22	165.5 ± 16
Nisoxetine	6.1 ± 1.1	7.6 ± 1.0	2.6 ± 0.9*
Cocaine	1993 ± 198	1906 ± 158	1282 ± 95*
[^3^H]WIN35428 Binding IC_50_ (nM)			
Nisoxetine	25.4 ± 3.7	29.8 ± 5.0	24.9 ± 2.9
Cocaine	35.5 ± 4.3	72.2 ± 10.7*	67.4 ± 6.2**

Data is presented as mean ± S.E.M. values from five to thirteen independent experiments. *p < 0.05, unpaired student’s *t*-test compared to WT hNET, **p < 0.01, unpaired student’s *t*-test compared to WT hNET.

## Data Availability

Data will be made available on request.

## Abbreviations:

cART: Combination antiretroviral therapy
BBB: Blood brain barrier
DA: Dopamine
DAT: Dopamine Transporter
hNET: Human norepinephrine transporter
Tat: Transactivator of transcription
3,4-[7-3H]: [^3^H]DA Dihydroxyphenylethylamine
V_max_: Maximal velocity
